# Congenital feeding response to a novel prey in a Mexican gartersnake

**DOI:** 10.7717/peerj.8718

**Published:** 2020-03-05

**Authors:** Javier Manjarrez, Constantino Macías Garcia, Hugh Drummond

**Affiliations:** 1 Facultad de Ciencias, Universidad Autónoma del Estado de México, Toluca, Estado de México, México; 2 Instituto de Ecología, Universidad Nacional Autónoma de México, Ciudad Universitaria, Ciudad de México, Mexico

**Keywords:** Neonates, Thamnophis, Crayfish, Chemosensory response, Ingestive response, Feeding niche, Behavioral response

## Abstract

In this study, we explored chemosensory, ingestive and prey-catching responses of neonate Mexican Black-bellied Gartersnakes (*Thamnophis melanogaster*) to crayfish (*Cambarellus montezumae*). By comparing snakes from a recently discovered crayfish-eating population and a typical non-crayfish-eating population, we asked which behavioral components change as a species enlarges its feeding niche. In the crayfish-eating population chemosensory responsiveness to crayfish was not enhanced but its heritability was higher. Neonates of both populations showed similar preference for freshly-molted versus unmolted crayfish, and whereas the tendency to ingest both crayfish stages remained stable between ages 15 and 90 days in the non-crayfish-eating population, in the crayfish-eating population it actually decreased. Techniques to catch and manipulate molted crayfish were similar in the two populations. We discuss the possibility that there is no increase in the behavioral response to eat crayfish by the neonates of the crayfish-eating populations, possibly due to the absence of ecological and spatial isolation between the two *T. melanogaster* populations. The crayfish ingestion in some population of *T. melanogaster* can be explained by environmental differences between populations, or by recent origin of crayfish ingestion in *T. melanogaster*.

## Introduction

Intraspecific geographic differences in snake diets have been associated with behavioral microevolution within species, and geographical variation in selection on feeding (Arnold, 1992; De Queiroz, Henke & Smith, 2001; Tuttle & Gregory, 2009). Congenital, chemically based preferences for different prey taxa have been widely demonstrated in several *Thamnophis* species (Burghardt, 1970; Burghardt, 1975; Burghardt, 1992). Newborn snakes respond to species-characteristic prey stimulus with high tongue-flick incidence and predatory strikes (Burghardt, 1993), and these behaviours have a heritable basis (Brodie Jr & Garland, 1993) with low to moderate heritabilities (0.04 to 0.36; Arnold, 1981a; Arnold, 1981b), although they may show different developmental trends (Burghardt, Layne & Konigsberg, 2000). Adaptive plasticity was documented when the chemically based congenital preference of Mexican black-bellied garter snakes for a dangerous species of sympatric leech declined after experiencing its defences (Drummond & Macías-García, 1995).

Thamnophiini (Natricinae) snakes normally consume soft prey such as fish, amphibians, slugs, and earthworms (Arnold, 1993; Rossman, Ford & Seigel, 1996), so predation on hard items could be select for behavioral and morphological adaptation (Dwyer & Kaiser, 1997; Rossman, 1963). Crayfishes are a difficult prey for snakes because they have a hard exoskeleton which is soft for only a few hours after molting (Godley, McDiarmid & Rojas, 1984). Generally, snakes must capture and manipulate crayfishes only when they are a soft and vulnerable prey (Gibbons & Dorcas, 2004; Godley, 1980; Mushinsky, Hebrard & Vodopich, 1982). This is exemplified by variation in the predatory behavior of snakes of the closely related genera *Regina* and *Lyodytes* (previously *Regina*) on crayfish (Godley, 1980; Mushinsky, Hebrard & Vodopich, 1982; Gibbons & Dorcas, 2004). *Regina septemvittata* (Queen Snake) and *R. grahamii* (Graham’s Crayfish Snake) capture freshly molted individuals only (Hall, 1969; Mushinsky & Hebrard, 1977; Godley, McDiarmid & Rojas, 1984), seizing them by the abdomen or head (Hall, 1969; Godley, McDiarmid & Rojas, 1984) without a significant preference for the direction of ingestion. *Lyodytes alleni* (Striped Crayfish Snake) and *L. rigida* (Glossy Crayfish Snake) also feed on hard, unmolted crayfish (Franz, 1977), and they capture all crayfish by the abdomen and constrain or press them against the substrate to ingest, preferably starting with the abdomen (Godley, 1980; Godley, McDiarmid & Rojas, 1984; Myer, 1987). In addition, the two pairs of *Regina* and *Lyodytes* species differ in the shape of their dentition and skulls (Myer, 1987; Fontenot, Platt & Dwyer, 1993; Dwyer & Kaiser, 1997). Mexican Black-bellied Gartersnakes are within the well-defined monophyletic group of garter snakes, whereas *Regina* and *Lyodytes* are polyphyletic with respect to other thamnophiines (Alfaro & Arnold, 2001; De Queiroz, Lawson & Lemos-Espinal, 2002; Guo et al., 2012; McVay & Carstens, 2013). This suggests that crayfish ingestion has probably arisen independently among *Regina* and *Lyodytes* species via evolutionary convergence associated with the ingestion of soft versus hard crayfish.

Crayfish ingestion is rare in *Thamnophis* spp. Although foraging in and around aquatic habitats is common, extensive field studies of the 31 species failed to reveal any instance except in *T. proximus*. In these cases crayfish made up 0.8% of stomach contents. (Rossman, Ford & Seigel, 1996; Hampton & Ford, 2007). However, in a population of the Mexican Black-bellied Gartersnake (*Thamnophis melanogaster*), which forages on soft-bodied prey such as fish, tadpoles, and leeches (Drummond, 1983), 35% of prey were crayfish *Cambarellus montezumae* (Manjarrez, Macías García & Drummond, 2013). The gartersnake is extensively sympatric with the crayfish throughout its range, but eats it only in a 6,000 km^2^ area straddling the back-to-back headwaters of the rivers Tula and Lerma, an area comprising only 3.0% of the area of sympatry (Manjarrez, Macías García & Drummond, 2013). This pattern has been interpreted as invasion of a niche novel to *Thamnophis* spp. and possibly in process of expansion through the Tula and Lerma basins, which provides an opportunity to study the early stages of microevolution toward the incorporation of a new prey in the diet (Arnold, 1981a). The 76 crayfish recovered from snake stomachs had been eaten within 24 h of molting, when the exoskeleton is still soft (Manjarrez, Macías García & Drummond, 2013), but chemosensory tests on wild-caught snakes suggested that freshly-molted and unmolted (hard) crayfish were both recognized as prey and readily attacked (Lozoya, 1988).

The Mexican black-bellied garter snake’s incorporation of a new prey into its diet in the headwaters of two adjacent basins is likely to involve two distinct processes whose relative importance in ecological divergence is the subject of much debate: microevolution (Arnold, 1981a) and phenotypic plasticity (West-Eberhardt, 2003). In garter snakes both are plausible.

In this study, we tested for a microevolutionary response of the crayfish-eating population of black-bellied garter snakes toward crayfish (*C. montezumae*). We compared inexperienced neonates born in captivity from crayfish-eating populations with neonates from non crayfish-eating populations for their chemosensory arousal, ingestion of crayfish tissue, and capture and manipulation of freshly-molted (soft) crayfish. We predicted neonates from the crayfish-eating populations would show tendencies in the direction of *Regina* species that specialize in eating soft crayfish. In relation to newly moulted *C. montezumae*, neonates from the crayfish-eating populations should show greater chemosensory and ingestive responsiveness, greater heritability of chemosensory responsiveness, ontogenetic stability or increase in ingestion of pieces, elevated predatory responses, and more efficient capture and manipulation. Presence of any of these effects would imply microevolution at an early stage of niche expansion, while absence of all of them could imply that niche expansion did not gain its initial impetus through microevolution.

## Materials & Methods

### Subjects

Neonates were born in captivity to gravid females captured at a crayfish-eating (C) sites (Acambay) and non crayfish-eating (NC) sites (El Cerrillo and San Pedro Tlaltizapán; Table S1), 90 km apart and all within the area of snake-crayfish sympatry where both species are continuously distributed (Lozoya, 1988; Manjarrez, Macías García & Drummond, 2013). Although crayfish ingestion in only some locations can be explained by subtle environmental differences between localities, a sampling suggested that, crayfish are more abundant in sites where snakes do not eat them that in where they do (Manjarrez, Macías García & Drummond, 2017). We collected snakes found basking on the ground or under rocks and in all available shelters and microhabitats. After capture, the pregnant females were transported to the Evolutionary Biology Laboratory at the “Universidad Aútonoma del Estado de México”. All snakes were collected under a scientific collecting permit issued by the federal institution (Secretaría de Medio Ambiente y Recursos Naturales, FAUT-0188). Snakes were housed at an ambient room temperature of 20–25 °C and a natural light–dark photoperiod, just 28 km from the nearest study population. Gravid females were individually housed in glass tanks (51 × 26 × 28 cm) containing water dishes and clay shelters. They were fed once a week with live fish until parturition. Neonates were removed from the parental cage 10–14 h after birth, sexed by eversion of male hemipenes, weighed and measured (snout-vent length), and individually housed in plastic cages (21 × 10 × 8 cm) with a water bowl and a paper substrate. Neonate cages were housed under similar environmental conditions to those of their mothers.

From birth to two weeks, neonates were not fed because they are born with a yolk reserve (Arnold, 1977; Arnold, 1992). At 14-days-old, they were exposed to a chemoreception test and an ingestion test at 15–21 days before being fed live fish ad libitum in their water bowls through day 83; at 90–96 days they had a second ingestion test. To ensure the reliability of the observations, all tests were carried out by the same observer. Test were performed at laboratory temperatures of 20−25 °C; afterwards all snakes were maintained in the laboratory until they could be returned to the field.

### Chemosensory test

We measured chemosensory responses in an aquatic environment of 253 neonates (14 days old) born to 34 females from NC localities (body weight of neonates: 1.75 ± 0.40 g), and 65 neonates born to 15 females from C localities (body weight of neonates: 2.02 ± 1.05 g; Data S1). The decision to perform the test on samples differing in number of neonates, with unbalanced sex and litters, was made in response to the difficult and unpredictable availability of gravid snakes from NC and C localities. Each neonate was transferred to a plastic box (20 × 13 ×  9 cm) filled with distilled water to a depth of 1.5 cm. Next day, the lid was removed and a cotton-tipped swab was slowly introduced to the box until its submerged tip was one cm in front of the snake’s snout (without touching it). Each snake was exposed successively in random order at 20–40 min interval to three aquaeous stimuli on swabs prepared in the manner of Arnold (1978); Arnold (1981b): newly-molted crayfish, non-molted crayfish, and a distilled water control. Crayfish-scented swabs were prepared by individually immersing and turning 15-cm wood cotton-tipped swabs in water with crayfish (volume 1:1) for 30 s until they were saturated. Control swabs were prepared immersed in plain distilled water. All swabs were prepared within an hour of each other and then frozen in airtight jars. Swabs were defrosted 30 min before each test. Swabs were presented underwater with a range of 20–40 min between each swab presentation (Waters & Burghardt, 2005; Waters & Burghardt, 2013) because *T. melanogaster* forages exclusively underwater (Drummond, 1983). Each swab was used once and then discarded.

Each chemical stimulus was presented to an individual for a maximum of 60 s.We counted the tongue flicks that touched the swab during this time or until the snake struck the swab, and measured the latency to strike in seconds (Drummond & Macías-García, 1995; Waters & Burghardt, 2005; Waters & Burghardt, 2013). A tongue-flick attack-score (TFAS) was calculated for each trial (Cooper Jr & Burghardt, 1990): *TFAS* =* TF + (60 - L*); where *TF* is the number of tongue-flicks for any stimulus for each individual and *L* is the latency to the strike in seconds. TFAS merges both latency to strike and tongue-flicks, as an index of response strength to a chemical stimulus with the highest values when the snake attacks the swab. This test is highly reliable and repeatable (Burghardt, Layne & Konigsberg, 2000; Waters & Burghardt, 2005; Waters & Burghardt, 2013).

### Statistical Analysis of Chemosensory Test

TFAS was transformed with the natural logarithm (ln-TFAS) to meet assumptions of normally-distributed residuals and homoscedasticity (Brodie Jr & Garland, 1993; Sokal & Rohlf, 1995). After transformation of the data, we maked three different sets of analysis. First, we used a generalized linear mixed model (ANOVA) with TFAS toward each stimulus being repeated measures variables. For each stimulus, the between groups variables were sex, litter (considered as random factors) and population type (C/NC) as fixed factor. After, we performed a planned one-way ANOVA for each group of localities (C and NC) compared the mean ln-TFAS by litter for each crayfish stimulus with the control. Later, to obtain a measure of responsiveness to soft crayfish versus the control we subtracted the freshly-molted crayfish mean litter ln-TFAS from the control scores (Brodie Jr & Garland, 1993). This difference is referred to as “relative response to control.” We compared the responses on control difference scores between C and NC using two-sample Student-*t* tests. To compare the stimulus preference for soft crayfish between populations, we calculated the difference between the mean litter ln-TFAS recorded for soft crayfish from the hard crayfish. This difference is referred to as “relative response to hard crayfish.” Finally, we compared the proportion of neonates that attacked to soft and hard crayfish swabs using a Chi-square contingency test, while the number of attacks toward each stimulus was analyzed by a *G* contingency test.

Heritability, in the narrow sense (*h*^2^ = ratio of additive genetic variance to total phenotypic variance) of the log-transformed TFAS for each stimulus was estimated by intraclass correlation, obtained from a one-way analyses of variance (ANOVA) as full siblings families (Falconer, 1989). Although multiple paternity has been found in some *Thamnophis* species (Schwartz, McCracken & Burghardt, 1989; Barry, Weatherhead & Philipp, 1992), in *T. melanogaster* there is no evidence of multiple paternity (Wusterbarth et al., 2010). Even in the existence of multiple paternity, most siblings families are, on average, nearly full-sibs (Schwartz, McCracken & Burghardt, 1989; Garner & Larsen, 2005), therefore the full sibling design is appropriate because it produces conservative estimates (Brodie Jr & Garland, 1993). Furthermore, estimates of heritability can be inflated by maternal effects (Falconer, 1989), but traits are buffered against the most conspicuous type of maternal influence (Arnold, 1981c; Arnold & Peterson, 2002; Manier, Seyler & Arnold, 2007). The heritability based on a single individual measure, without repeatability measures, includes whole component to the special environmental variance (Brodie Jr & Garland, 1993), however this estimated heritability for a single trial could be 0.82 times the heritability estimate for the average of two trials, as demonstrated for *T. radix* (Arnold & Bennett, 1984). Significance of heritability was estimated by the Jacknife parametric technique (Sokal & Rohlf, 1995) with 95% confidence limits, using the Student*’s t* test. We report mean values ± 1 *SD*.

### Ingestion test

One day after performing the chemosensory test, at age 15 days an ingestion test was applied to the above neonates in their individual home cages, and 75 additional (untested) neonates, totalling 305 subjects from 41 females from NC localities and 88 subjects from 19 females from C localities. The number of neonates in the ingestion test was greater than the number used in the chemosensory test because our initial protocol of chemosensory test was performed by presenting the swabs out of the water. The out-of-water swab chemosensory test results are not included in the analysis. We consider that adding more neonates to ingestion test would not confound our capability to determine genuine differences between chemosensory and ingestion snakes’response, because both tests, are different behavioural responses. To compare congenital preferences to eating crayfish, we recorded neonates’ responses to a set of three food stimulus presented in a terrestrial environment: Soft crayfish (within 6 h of molt), hard unmolted crayfish, and fish (*Xiphophorus variatus*, to test for hunger) were frozen in airtight bottles, defrosted 20 min before testing (Arnold, 1978; Arnold, 1981c) and cut into 0.3-g pieces which were placed on glass slides (2.6 × 7 cm). We used small pieces to avoid satiation (Arnold, 1977; Arnold, 1981c; Macias Garcia & Drummond, 1988). At the start of the test snakes from NC localities and C localities weighed 1.75 ± 0.40 g and 2.02 ± 1.05 g, respectively, on average.

On days one and seven, we inserted a piece of fish in a corner of the home cage to verify that the neonates were hungry during the test (Arnold, 1978; Arnold, 1981a). On days two through six, we inserted a piece of soft crayfish in one corner and a piece of hard crayfish in the other, alternating the position on successive days. Twenty-four hours after insertion, we recorded ingestion and removed uneaten pieces. The ingestion of fish pieces and other aquatic prey offered outside of water has been previously demonstrated on newborns *T. melanogaster* (Macias Garcia & Drummond, 1988; Drummond & Macías-García, 1995).

From ages 15 to 83 days the subjects were kept in their individual home cages with weekly ad libitum access to live fish in their water bowls. At 90 days, the survivors, comprising 213 neonates of 37 females from NC localities and 70 neonates of 17 females from C localities, were re-tested following the same protocol, to detect change in the preference between soft and hard crayfish. In this way, these neonates had two ingestion tests (15 and 90 days old).

#### Statistical Analysis of Ingestion Test

We calculated the mean number of ingested pieces per litter of soft and hard crayfish. We compared the mean scores for soft crayfish from C versus NC populations using one-sample Student-*t* tests. Finally, we calculated a preference score for soft crayfish versus hard crayfish by subtracting the average number of ingested pieces per litter of soft crayfish from that of hard crayfish. We compared the preference score between C and NC populations using two-sample Student-*t* tests. To determine the variability of neonates to ingest prey during the five days of the crayfish ingestion test, we applied a two-way ANOVA (presentation day and litter) for each of the *T. melanogaster* populations. We compared with Chi-square contingency test, the proportion of neonates in C and NC populations who ingested only soft crayfish, only hard crayfish, both, or neither. Also, with Chi-square the hunger level was explored by analyzing the number fish pieces ingested by neonates on days 1 and 7 of the test and with Student*-t* test we compared the weight and length of C versus NC neonates.

### Predatory behavioral test

To compare effectiveness of C and NC snakes at capturing and manipulating soft crayfish (within 6 h of moulting) at ages 102-130 days, we observed the predatory behavior of 15 C juveniles and 10 NC juveniles, all of them single representatives of 25 laboratory-born broods used in chemosensory and ingestion tests .

After seven days without food, each snake was placed in a glass tank (51 × 26 × 28 cm) containing a 25 × 30 × 10 cm deep pool of water in a sand base. Next day, after gently introducing one-two live soft crayfishes (0.61 ± 0.14 g) to the experimental tank we registered all attacks (projection of open jaws toward crayfish) during 20 min. When successful, we noted the body part seized and from which ingestion began (thorax or abdomen), prey-handling time (between capture and swallowing), and the methods of searching for and subduing prey (behavioral categories as in Drummond, 1983).

### Statistical analysis of predatory behavioral test

We described the percentages to attacks to soft crayfis and compared prey-handling time from C versus NC populations and using a Mann–Whitney-U. To assess from which ingestion began (thorax or abdomen of the crayfish), we using a binomial tests.

This study received the approval of field permit (Secretaria del Medio Ambiente y Recursos Naturales #07164) and the ethics committee of the Universidad Autónoma del Estado de México (Numbers 3589/2013SF, 4047/2016SF, 4865/2019SF). All subjects were treated humanely on the basis of guidelines outlined by the American Society of Ichthyologists and Herpetologists (ASIH, 2004).

## Results

### Chemosensory test

Separate repeated measures ANOVAs showed that there was no effect of sex and litter at each stimulus on tongue-flick attack score. However, there was an effect of sex only in control stimulus (Table S2).

**Figure 1:**
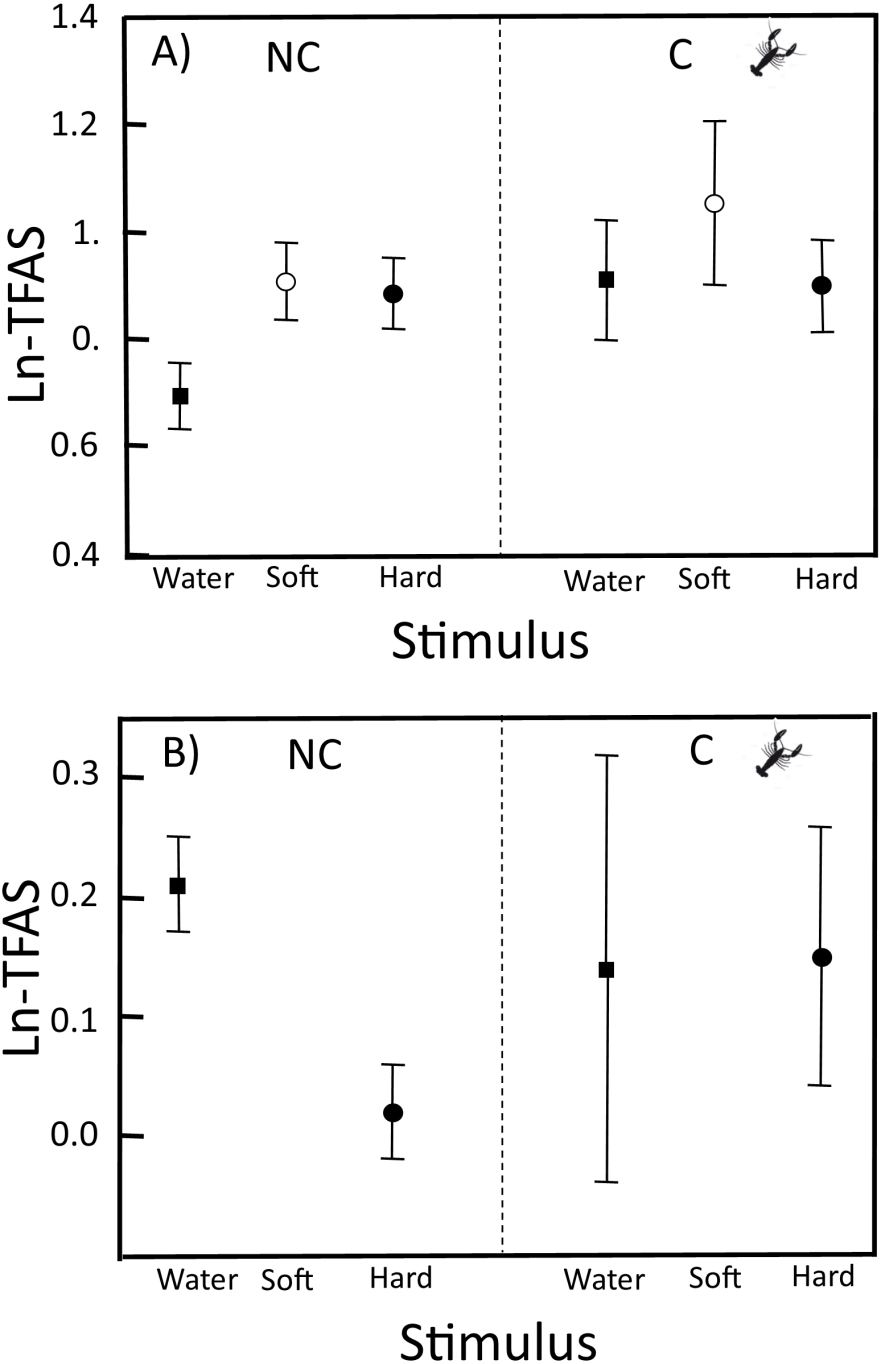
TFAS to swabs with distilled water, soft crayfish, and hard crayfish stimulus in clutches from non-crayfish-eating and crayfish-eating *T. melanogaster* populations. (A) Mean ln-TFAS (±1 *SE*) and (B) Relative response to control (water) and relative response to hard crayfish. Thirty-four and 15 clutches from non-crayfish-eating (NC) and crayfish-eating (C) *T. melanogaster* populations, respectively.

In the neonates from NC localities, ln-TFAS for soft crayfish (0.91 ± 0.36) and hard crayfish (0.88 ± 0.35) were 1.3 and 1.2 times higher, respectively, than for the control water (0.69 ± 0.33; *F*_2,99_ = 3.28, *P* = 0.04; Fig. 1). In neonates of C localities, the ln-TFAS for soft crayfish (1.05 ± 0.47.) and hard crayfish (0.90 ± 0.25) were similar to the control (0.91 ± 0.32; *F*_2,42_ = 0.52, *P* = 0.60; Fig. 1A). The relative response for control was similar between C (0.14 ± 0.54) and NC neonates (0.21 ± 0.25; Student*-t*_47_ = 0.53, *P* = 0.70; Fig. 1B). The relative response for hard crayfish was also similar between C (0.15 ± 0.33) and NC individuals (0.02 ± 0.25; Student*-t*_47_ = 1.22, *P* = 0.11; Fig. 1B).

The number of attacks was low in both groups of neonates. Only 3.1% of neonates from C and 11.5% from NC attacked a swab. Of C localities, only two neonates attacked a swab with soft crayfish stimulus and did not attack other odors, while NC neonates attacked in similar percentages to soft and hard crayfish swabs (6.3% and 5.2%, respectively; *X*^2^ = 0.31, *df* = 1, *P* = 0.58), but did not attack the control-water swabs. Attacks were only recorded towards hard and soft crayfish swabs. The number of attacks towards soft crayfish swabs (*N* = 328) was higher than hard crayfish swabs (*N* = 151; *G* = 5.34, *df* = 1, *P* = 0.02) in both *T. melanogaster* populations.

In C neonates, the heritability of chemosensory response to soft crayfish (1.00 ± 0.33) was greater than NC neonates (0.64 ± 0.28), while heritability to hard crayfish was lower (0.13 ± 0.48) than NC neonates (0.71 ± 0.35; Table 1). In the neonates from C localities, heritability to soft crayfish was higher than hard crayfish and control water (0.15 ± 0.40), while neonates from NC had heritability values that were similar for soft and hard crayfish (Table 1).

**Table 1:** Heritability (*h*^2^) of chemosensory response (ln-TFAS) of neonates *T. melanogaster* to crayfish stimulus in C and NC neonates. Heritability of the log-transformed TFAS for each stimulus estimated by intraclass correlation, obtained from a one-way analyses of variance as full siblings families.

**Stimuli**	**C**			**NC**		
	15clutches, 65 neonates			34clutches, 253 neonates		
	***h***^**2**^±*SE*	***p***	**95% Confidence limit**	***h***^**2**^±*SE*	***p***	**95% Confidence limit**
Soft crayfish	1.00 ± 0.33	<0.001	0.71–1.95	0.64 ± 0.28	>0.05	−0.01–0.95
Hard crayfish	0.13 ± 0.48	>0.05	−0.79–0.99	0.71 ± 0.35	>0.05	−0.02–1.17
Water	0.15 ± 0.40	>0.05	−0.67–0.83	0.95 ± 0.29	<0.001	0.89–1.91

### Ingestion test

A Generalized linear mixed model (ANOVA of separate repeated measures, showed that there was no effect of litter and sex (random factors) at each type of crayfish and fish on number of ingested pieces (Table S2).

#### Fifteen days old

**Figure 2:**
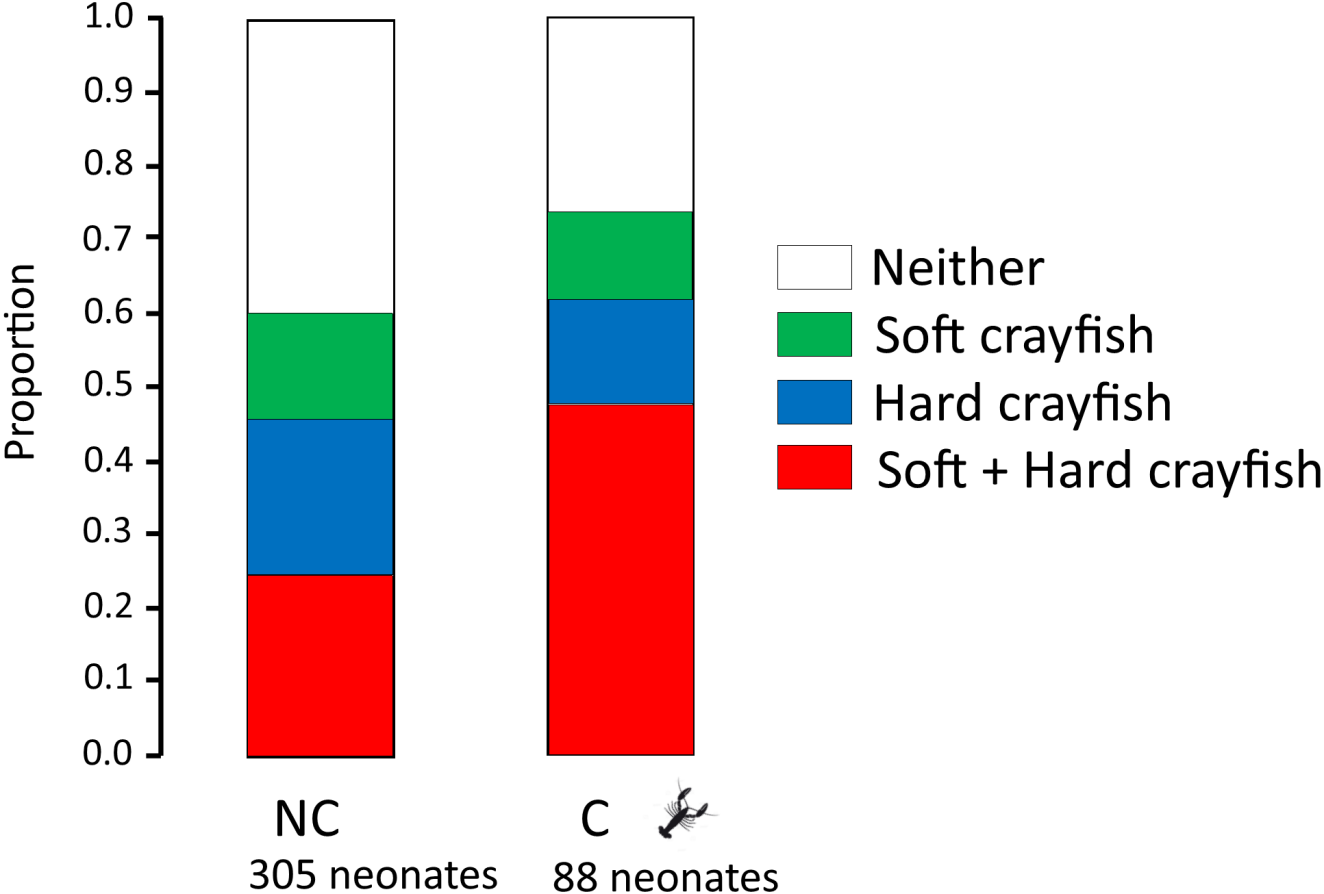
Proportion of neonates who ingest only soft crayfish, only hard crayfish, both, or neither, from both *T. melanogaster* populations. NC: Non-crayfish-eating neonates, C: crayfish-eating neonates.

**Figure 3:**
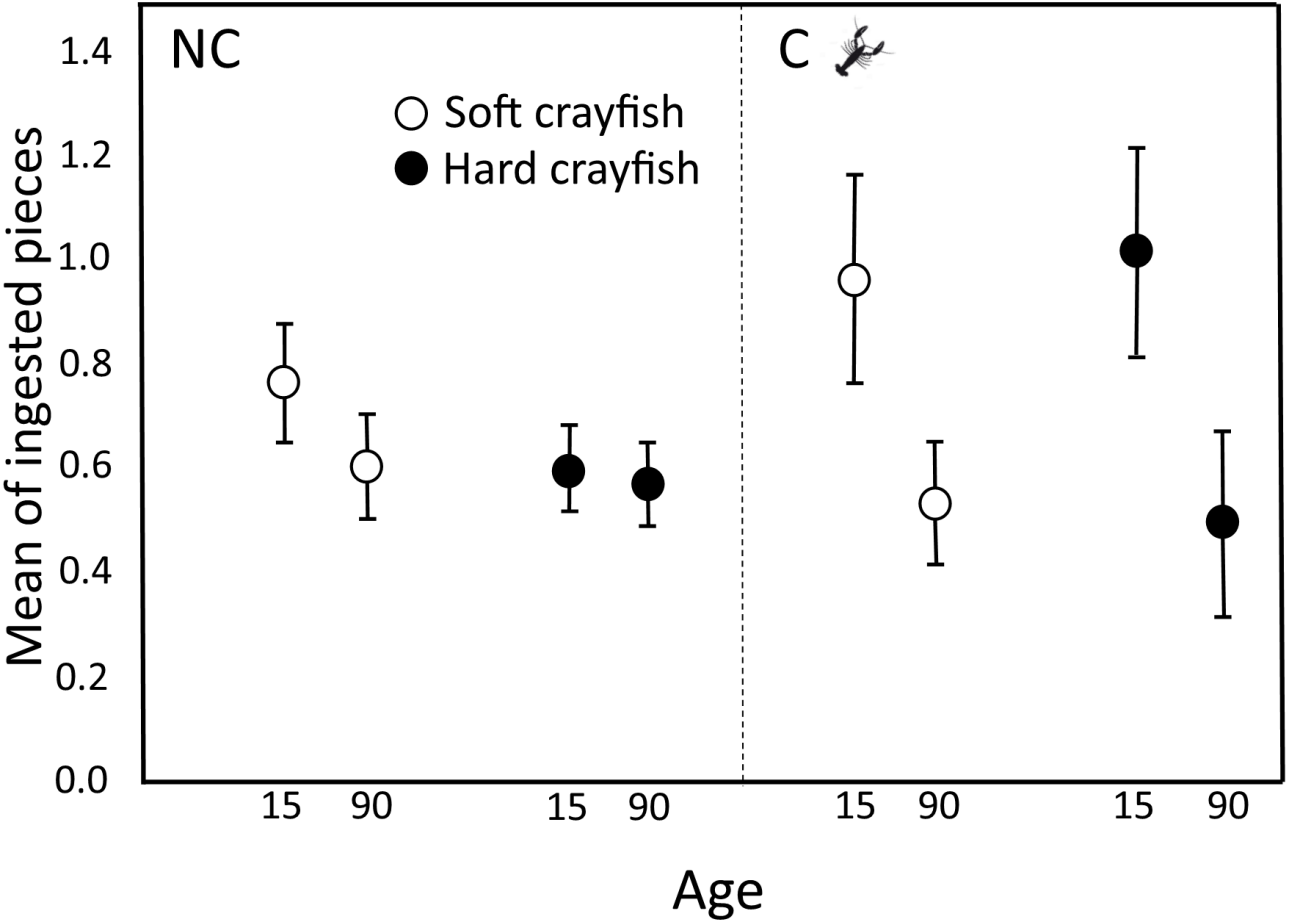
Ingested pieces of soft and hard crayfish by 15- and 90-day-old neonates from non-crayfish-eating (NC) and crayfish-eating (C) *T. melanogaster* populations. Mean number (±1 *SE*) of ingested pieces per clutch.

The proportion of neonates who ingested only soft crayfish, only hard crayfish, both, or neither, was different between both *T. melanogaster* populations (*X* ^2^ = 12.08, *df* = 3, *P* = 0.009; Fig. 2). Soft crayfish was ingested for a mayor proportion of C neonates (61%) than NC neonates (46%; *X*^2^ = 4.5, *df* = 1, *P* = 0.03). Similarly, hard crayfish was ingested in greater proportion by C neonates (60%), than NC neonates (39%; *X* ^2^ = 7.99, *df* = 1, *P* = 0.005). Mean number of ingested pieces per clutch of soft crayfish was similar between both *T. melanogaster* populations (Student*-t*_58_ = 0.90, *P* = 0.18; Fig. 3). The preference score for soft crayfish also was similar between C (0.16 ± 0.51) and NC individuals (0.04 ± 0.60; Student*-t*_58_ = 1.15, *P* = 0.87).

A difference in hunger level may explain the higher ingestion of both soft and hard crayfish by neonates from C localities (Fig. 2). In days 1 and 7, neonates from C populations ingested more fish pieces than neonates from NC (day 1, 79% vs 41%; *X*^2^ = 26.6, *df* = 1, *P* = 0.0001), but on day 7 this difference did not reach significance (day 7, 60% vs 46%; *X*^2^ = 3.44, *df* = 1, *P* = 0.07). The higher hunger level may also be due to the fact that C neonates were 15.5% heavier (2.02 ± 1.05 g) and 8.5% longer (15.4 ± 2.15 cm SVL) than NC (1.75 ± 0.40 g; Student*-t*_348_ = 2.82, *P* = 0.004; 14.2 ± 1.59 cm SVL; Student*-t*_391_ = 4.38, *P* = 0.0001).

**Figure 4:**
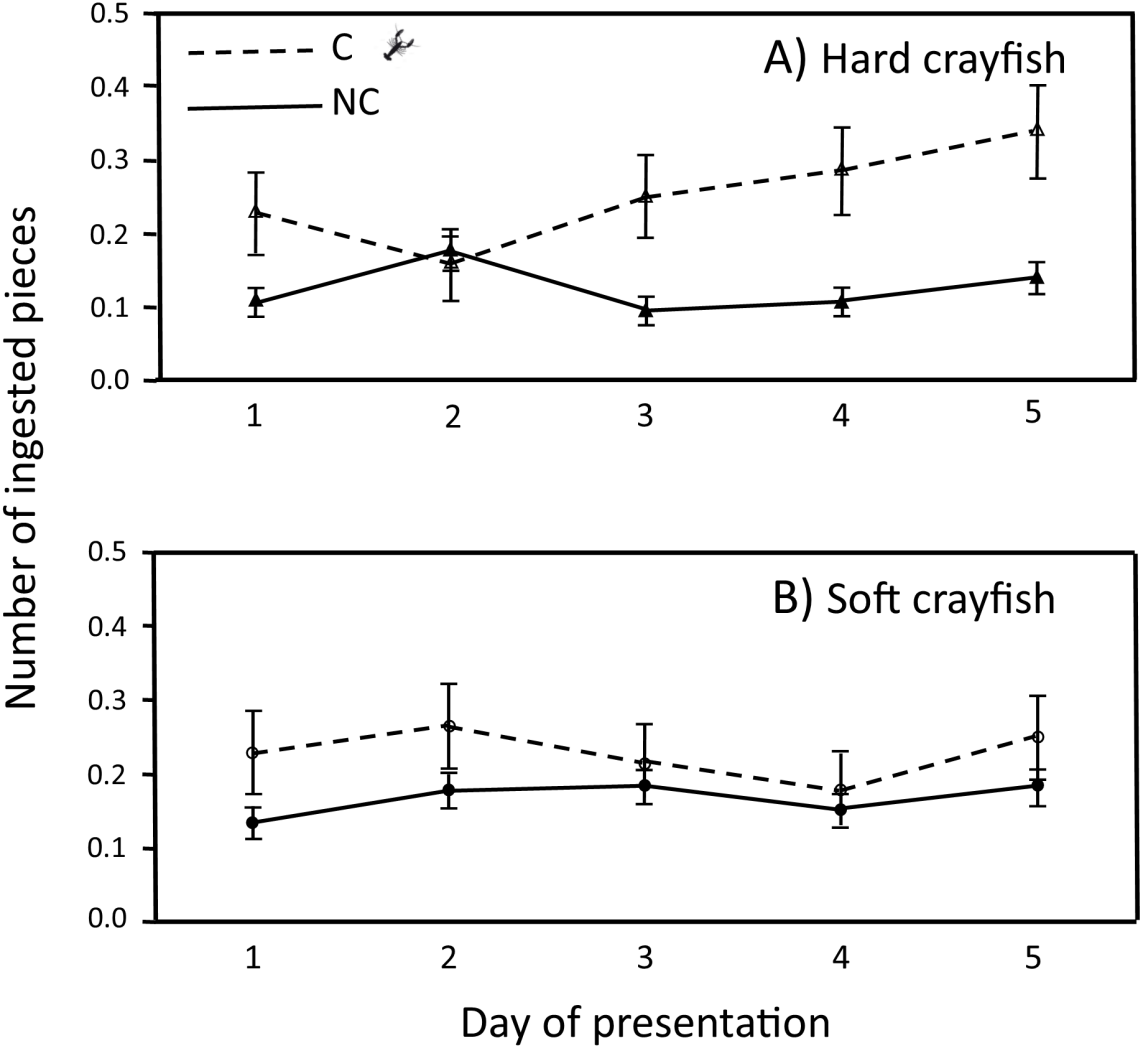
Ingestion frequency (*X* ± 1 *SE*) of (A) hard and (B) soft crayfish during the five days of presentation by neonates from C and NC populations.

**Figure 5:**
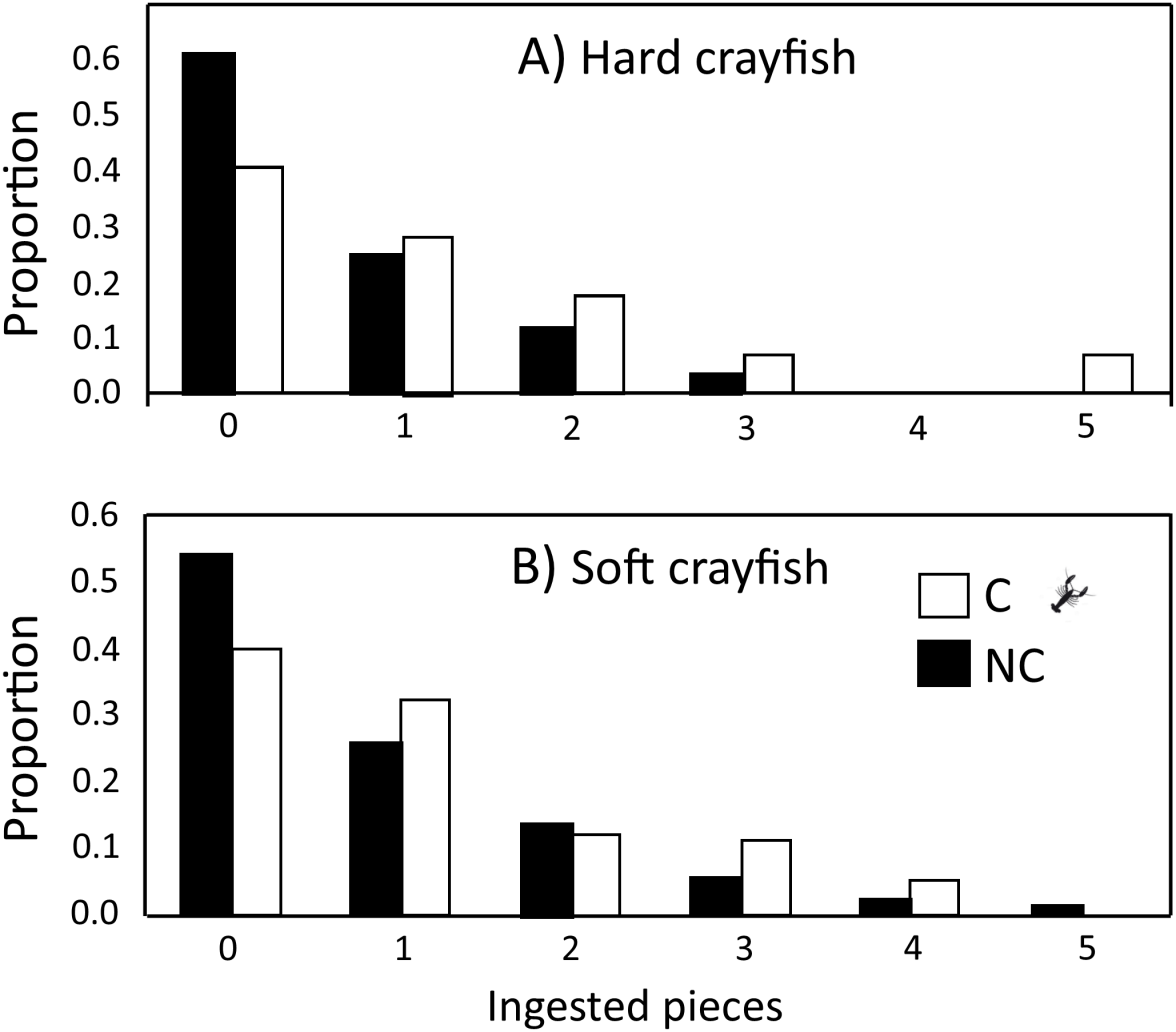
Frequency distributions of number of (A) hard and (B) soft crayfish pieces ingested during the five days of presentation neonates from C and NC populations.

The ingestion frequency of soft crayfish did not change during the five days of the test for C (*F*_4,83_ = 0.35, *P* = 0.84), and NC neonates (*F*_4,300_ = 0.92, *P* = 0.45; Fig. 4). The same was true for the hard crayfish pieces in C neonates (*F*_4,83_ = 1.66, *P* = 0.16), but NC neonates showed significant variation between days (*F*_4,83_ = 2.66, *P* = 0.03; Fig. 4). In all cases, the frequency distributions for number of crayfish pieces ingested during the five days of presentation presented unimodal distribution patterns, with mode of zero ingestions (Fig. 5).

#### Ninety days old

The proportion of neonates that ate only soft crayfish, only hard crayfish, both, or neither was similar between 15 and 90 days old for both *T. melanogaster* populations (C, *X*^2^ = 3.3, *df* = 3, *P* = 0.32; NC, *X*^2^ = 5.9, *df* = 3, *P* = 0.15).

For NC neonates, the mean number of ingestions per clutch of soft crayfish did not change between 15 (0.76 ± 0.11) and 90 days old (0.61 ± 0.10; Dependent Student*-t*_40_ = 1.65, *P* = 0.11; Fig. 3), nor did it change for hard crayfish (15 d old, 0.60 ± 0.08; 90 d old, 0.58 ± 0.08; Student*-t*_40_ = 0.93, *P* = 0.32; Fig. 3).

For C neonates, the preference to ingest pieces of crayfish changed between 15 and 90 days old. At 90 days old, neonates ingested half the soft crayfish (0.54 ± 0.12 pieces per clutch) that 15-day-olds ingested (1.17 ± 0.20; Student*-t*_18_ = 3.8, *P* = 0.002; Fig. 3), and the same decrease applied to hard crayfish (15 d old, 1.02 ± 0.20; 90 d old, 0.50 ± 0.18; Student*-t*_18_ = 3.1, *P* = 0.003; Fig. 3).

### Predatory behavioral test

Both C and NC snakes cruised, dove and crawled on the underwater substrate, and occasionally approached and attacked crayfish, including 50% of 10 C snakes and 27% of 15 NC neonates, usually after bumping their snouts against them. Two C snakes and three NC snakes captured crayfish, by the thorax or abdomen in similar proportions (Binomial test, *P* = 0.36), and began ingestion at the capture point. Handling time was also similar between C (217 ± 32.5 s, *n* = 3 ingestions) and NC neonates (243 ± 19.3 s, *n* = 2; Mann–Whitney- *U* = 56.7, *p* = 0.78). Neonates of both *T. melanogaster* populations, attacked with open jaws holding the crayfish and swallowing with the movements of the skull bones.

## Discussion

Manjarrez, Macías García & Drummond (2013) reported a crayfish-eating novelty of Mexican Black-bellied Gartersnake from eats crayfish only in a 3.0% of the area extensively sympatric and suggested that this novelty was a result of inherent preferences rather than prey availability. Our study suggests that *T. melanogaster* are born with an innate chemosensory and ingestion interest in both freshly-molted (soft) and unmolted (hard) crayfish, because are both recognized as prey and readily attacked. In general, laboratory tests do not support the prediction of greater innate chemosensory, ingestion and predatory behavioral response to only freshly-molted crayfish (*C. montezumae*) by C snakes *T. melanogaster*. In addition, C and NC neonates had a similar congenital chemosensory and ingestion response to both soft and hard crayfish, although the response and heritability to odors of soft crayfish were higher in C neonates. The tests also failed to demonstrate the existence of variation in techniques to capture and manipulate the soft crayfish between either *T. melanogaster* populations.

Due to the low response behavioral response to crayfish stimuli by the neonates *T. melanogaster*, the lack of clear results is understandable. Although *T. melanogaster* is considered as highly defensive and prone to attack at birth (Herzog & Burghardt, 1986; Herzog & Burghardt, 1988; Herzog, Bowers & Burghardt, 1989a; Herzog, Bowers & Burghardt, 1989b; Herzog, Bowers & Burghardt, 1992), this propensity was not observed in our study. For example, even from the crayfish-eating population, 97% of the snakes did not attack a swab. This is reasonable, because *T. melanogaster* is less responsive to chemical cues, even from high-preferred prey such as fish, than the generalist *T. sirtalis* (Ford & Burghardt, 1993). On the other hand, the criteria to consider *T. melanogaster* as highly defensive and prone to attack, is based on studies developed from the defensive and antidepredatory responses of this snake (Herzog & Burghardt, 1986; Herzog & Burghardt, 1988; Herzog, Bowers & Burghardt, 1989a; Herzog, Bowers & Burghardt, 1989b; Herzog, Bowers & Burghardt, 1992), a different behavior from the behavioral feeding response recorded of this study.

It is possible there is no increased food behavioral response to soft crayfish by neonates of the populations that eat crayfish. We know of no physical, environmental or genetic barrier between the rivers Lerma and Tula, which completely isolates both *T. melanogaster* populations. So it is likely that migration and gene flow can occur between both locations, especially for snakes and other reptiles in the trans-Mexican volcanic belt (Sunny et al., 2015; Sunny, González-Fernández & D’Addario, 2017; González-Fernández et al., 2018), where *T. melanogaster* is present. Crayfish ingestion in only some locations may possibly be explained by environmental differences between localities (Arnold, 1981a); for example, spatial–temporal availability of crayfish or differences in use of microhabitats by *T. melanogaster*. For example, crayfish availability influences the innate chemosensory responses to crayfish in the snake, *Lyodytes alleni* (Waters & Burghardt, 2013). However, a sampling during the rainy season (June to October) indicated a greater abundance of crayfish in the ponds where snakes do not eat crayfish than where they do eat crayfish (mean of three drags = 2.08 ± 3.03 crayfish / haul network of 2.8 m, mesh five mm, in 8 NC localities, versus 0.66 ± 1.2, in 9 C localities), although Student*-t* did not detect a significant difference (*P* = 0.23).

Another explanation could be that when snakes depend on the crayfish, the other usual prey are less available. However, this possibility has little support because Lozoya (1988) found no evidence of a temporary preference in the ingestion of fish by *T. melanogaster* in the different locations of the Lerma and Tula basins. He also reports that snakes that eat crayfish also feed on other types of prey in a higher percentage (65%) than the crayfish, which suggests that crayfish availability and ingestion is independent of the availability of other prey.

Alternatively, there may be increased food behavioral response to crayfish by C neonates, but it was not detected by our test protocol due that this food behavioral difference is small in magnitude and hardly detectable. This is possible when soft crayfish is not a profitable prey or have foraging disadvantages to the snake. The decreased ingestion of soft and hard crayfish at 90 days old by neonates in C populations suggests a learning process to recognize the crayfish as a non-profitable prey compared to fish with which neonates were fed from 15 to 90 days old. Also, the results of the predatory behavioral test, suggest that freshly-molted crayfish is not fully recognized as profitable prey by either *T. melanogaster* dietary morph. A small behavioral divergence in magnitude between both populations may be due to a recent origin, so that behavioral divergence for recognizing the crayfish has not yet been expanded into the surrounding geographical areas.

The ancestor of *Thamnophis* has existed in North America for at least 13 million years and its divergence as a genus occurred possibly two million years ago in Mexico (Alfaro & Arnold, 2001; De Queiroz, Lawson & Lemos-Espinal, 2002). The last geological events that altered river systems where *T. melanogaster* eat crayfish (Barbour, 1973) occurred two million years ago. For *T. elegans*, Arnold (1981a) estimated that food divergence for ingesting slugs occurred about 8,000 years ago assuming low intensity selection of 1.0%. The appearance of crayfish ingestion in *T. melanogaster* is possibly more recent than 8,000 years ago considering that high heritability values to freshly-molted crayfish (Table 1) cause a rapid evolution of food preference.

Neonates of both *T. melanogaster* populations responded similarly to both crayfish odors, although in C neonates the heritability was higher for freshly-molted crayfish. This suggests three possibilities: (1) Crayfish ingestion in *T. melanogaster* was an ancestral behavioral character, and only remains a homologous behavior in populations with similar ecological conditions to ancestral populations. This possibility can be explored with a study of the phylogeographic structure of both dietary crayfish populations, which helps explain the patterns of gene flow between populations (Foster & Cameron, 1996). (2) Neonates of both *T. melanogaster* populations are unable to recognize the difference between soft and hard crayfish. This possibility is supported by the results of this study and also for *R. grahamii, R. septemvittata*, and fish-eating species such as *Nerodia sipedon* (Burghardt, 1968). (3) The high and significant heritability to freshly-molted crayfish by *T. melanogaster* neonates supports the existence of microevolution toward the incorporation of a new prey in the diet through the divergence between the two *T. melanogaster* populations; a process that may be recent, as previously discussed.

After birth, experience with fish did influence ingestion response to both crayfish by C snakes *T. melanogaster*, but not for NC snakes. C 90-day-old neonates ingested less toward both soft and hard crayfish, conversely NC neonates continued to respond both crayfish (see Fig. 1).

Why did only C snakes at 90-days-old decrease ingestion of crayfish pieces compared to 15-days-old? This decrease can be the result of different processes. First, the reduction or disappearance of hunger level in *T. melanogaster* neonates. *Regina grahamii* and *R. septemvittata* have been shown that hunger level differences affects the chemosensory response to crayfish (Waters & Burghardt, 2005). In *T. melanogaster* there may be a similar phenomenon in response to an alternative prey (soft crayfish) because in specialist-species hunger may influence predator behavior (Ford & Burghardt, 1993) through the choice of suboptimal or alternative prey that snakes can use, such as crayfish (Arnold, 1993). Decreased ingestion of crayfish pieces may also be the result of ontogenetic change in food response to prey. Ontogenetic changes in prey preferences between neonates and juveniles for several *Thamnophis* species have been widely demonstrated and associated with dietary experience and maturation; however few studies report these changes in times as short as those of this study; 15-90 days old (reviewed in Rossman, Ford & Seigel, 1996). For *T. melanogaster*, the analysis of his diet through the Tula and Lerma basins has not detected an ontogenetic change in crayfish ingestion (Manjarrez, Macías García & Drummond, 2013). Other possibility is that innate chemosensory responses to prey can be modified by prey availability and that they do not necessarily result from maturation alone, as was demonstrated in the innate chemosensory response to crayfish by *Lyodytes alleni* (Waters & Burghardt, 2013). Newborn *R. alleni* feeds on dragonfly larvae to survive the critical period after birth when suitable sizes of crayfish, their preferred prey are not available. In this context, *T. melanogaster* would develop an innate chemosensory preference for a prey when it becomes finally available, months after the birth of the offspring. Finally, the degree to which dietary experience influences chemosensory response to prey stimulus depends on the species being studied (Burghardt, 1993). Species fed alternative prey types often develop chemosensory preferences for initially less-preferred prey (Waters & Burghardt, 2005).

Ingestion of crayfish by *T. melanogaster* could be the beginning of an evolutionary convergence with the two species that specialize in soft crayfish, *R. grahamii* and *R. septemvittata* (Hall, 1969; Mushinsky & Hebrard, 1977). In regards to *Regina* spp., Dwyer & Kaiser (1997) suggest that ingestion of soft prey (such as freshly-molted crayfish), is a precondition to specialization towards the ingestion of hard prey (such as non-molted crayfish). This hypothesis supports the idea of invasion of a novel food niche and convergence with *Regina* and *Lyodytes* in C *T. melanogaster* populations. In addition to this food convergence, C *T. melanogaster* populations have dentition and morphological head features that also subtly trend toward convergence with *R. grahamii* and *R. septemvittata* (Manjarrez, Macías García & Drummond, 2017).

## Conclusions

In conclusion, our analyses weakly support the prediction innate chemosensory, ingestion and predatory behavioral response by C snakes *T. melanogaster* associated with the ingestion of the freshly-molted crayfish, although the response and heritability to odors of soft crayfish were higher in C neonates. The weak tendency of behavioural feeding tendencies in the direction of *Regina* and *Lyodytes* could imply that niche expansion did not gain its initial impetus through microevolution possibly due to a recent origin without, so that behavioral divergence has not yet been expanded into the surrounding geographical areas.

Because the low response level to crayfish stimuli questions our results, a future exploration is required that includes within the protocol other prey stimuli known to be ingested by *T. melanogaster*, such as fish or tadpoles.

##  Supplemental Information

10.7717/peerj.8718/supp-1 Table S1 Capture location of gravid *T. melanogaster* females from crayfish-eating (C) and non-crayfish-eating (NC) field sites and the birth year of their clutches that were used in chemosensory, ingestion, and behavioral tests Clutch size is mean number of neonates by clutch ± 1 *SD*.

10.7717/peerj.8718/supp-2 Table S2 Generalized linear mixed models with separated repeated measures ANOVAs for TFAS and ingested pieces toward each stimulus by sex, litter (as a random factors) and population type (NC/C), as fixed factors of neonates *T. melanogaster.*

10.7717/peerj.8718/supp-3 Data S1 Raw data of neonates *Thamnophis melanogaster* Neonates of *Thamnophis melanogaste* r from crayfish-eating population and non-crayfish-eating population

## Additional Information and Declarations

### Field Study Permissions

The following information was supplied relating to field study approvals (i.e., approving body and any reference numbers): Secretaria de Medio Ambiente y Recursos Naturales approved the study (07164).

### Data Availability

The following information was supplied regarding data availability: The raw measurements are available in Data S1.
