# Supplementary material for: Congenital feeding response to a novel prey in a Mexican gartersnake
Source: PeerJ. 2020 Mar 5;8:e8718. doi: 10.7717/peerj.8718 (PMC7060902; doi:10.7717/peerj.8718)
Supplement: Table S1 — Clutch size is mean number of neonates by clutch ± 1 SD. [file peerj-08-8718-s001.docx]

Supplementary Table 1.

| **Year** | **C** | **clutches** | **clutch size** | **NC** | **clutches** | **clutch size** |
| --- | --- | --- | --- | --- | --- | --- |
| **1996** | Acambay | 4 | 13.0 ± 2.2 | El Cerrillo | 7 | 5.6 ± 2.0 |
| **1997** | Acambay | 3 | 5.1 ± 3.1 | San Pedro Tlaltizapan | 8 | 3.7 ± 2.9 |
| **1998** | Acambay | 3 | 10.8 ± 6.1 | San Pedro Tlaltizapan | 7 | 4.0 ± 0.7 |
| **1999** | Acambay | 9 | 6.3 ± 4.0 | San Pedro Tlaltizapan | 16 | 7.0 ± 4.5 |
|  |  |  |  | El Cerrillo | 3 |  |
