# Supplementary material for: Congenital feeding response to a novel prey in a Mexican gartersnake
Source: PeerJ. 2020 Mar 5;8:e8718. doi: 10.7717/peerj.8718 (PMC7060902; doi:10.7717/peerj.8718)
Supplement: Table S2 [file peerj-08-8718-s002.docx]

Supplementary Table 2.

| **Test** | **Stimulus** |  | ***df*** | ***F*** | ***P*** |
| --- | --- | --- | --- | --- | --- |
| **TFAS** | **Water** | Litter | 47 | 2.55 | 0.08 |
|  |  | Sex | 1 | 4.54 | **0.03** |
|  |  | Population (C/NC) | 1 | 1.99 | 0.15 |
|  | **Soft crayfish** | Litter | 47 | 1.6 | 0.19 |
|  |  | Sex | 1 | 2.15 | 0.14 |
|  |  | Population (C/NC) | 1 | 3.17 | 0.07 |
|  | **Hard crayfish** | Litter | 47 | 0.37 | 0.68 |
|  |  | Sex | 1 | 3.65 | 0.06 |
|  |  | Population (C/NC) | 1 | 0.01 | 0.90 |
| **Ingested pieces (Fifteen days old)** | **Fish** | Litter | 58 | 3.06 | 0.43 |
|  |  | Sex | 1 | 0.79 | 0.37 |
|  |  | Population (C/NC) | 1 | 6.78 | **0.01** |
|  | **Soft crayfish** | Litter | 58 | 3.37 | 0.41 |
|  |  | Sex | 1 | 0.18 | 0.67 |
|  |  | Population (C/NC) | 1 | 2.29 | 0.13 |
|  | **Hard crayfish** | Litter | 58 | 3.52 | 0.40 |
|  |  | Sex | 1 | 1.53 | 0.21 |
|  |  | Population (C/NC) | 1 | 17.45 | **0.00** |
